# Early influenza virus characterisation and vaccine effectiveness in England in autumn 2025, a period dominated by influenza A(H3N2) subclade K

**DOI:** 10.2807/1560-7917.ES.2025.30.46.2500854

**Published:** 2025-11-20

**Authors:** Freja CM Kirsebom, Catherine Thompson, Tiina Talts, Beatrix Kele, Heather J Whitaker, Nick Andrews, Nurin Abdul Aziz, Christopher Rawlinson, Rebecca E Green, Catherine Quinot, Nicholas Gardner, Elizabeth Waller, Alex Allen, Conall H Watson, Suzanna LR McDonald, Maria Zambon, Richard Pebody, Mary Ramsay, Katja Hoschler, Anika Singanayagam, Jamie Lopez Bernal

**Affiliations:** 1 Immunisation and Vaccine-preventable Diseases Division, UK Health Security Agency, Colindale, London, United Kingdom; 2 Respiratory Virus Unit (RVU), UK Health Security Agency, Colindale, London, United Kingdom; 3 Modelling Division, UK Health Security Agency, Colindale, London, United Kingdom; 4 NIHR Health Protection Research Unit in Respiratory Infections, Imperial College London, United Kingdom; 5 Epidemic & Emerging Infections Directorate, UK Health Security Agency, Colindale, London, United Kingdom; 6 Public Health Programmes Directorate, UK Health Security Agency, Colindale, London, United Kingdom; 7 NIHR Health Protection Research Unit in Immunisations, London School of Hygiene and Tropical Medicine, United Kingdom; * These authors contributed equally to this work and share last authorship.

**Keywords:** Influenza, A(H3N2), J.2.4.1, subclade K, vaccine effectiveness, test-negative design

## Abstract

Influenza A(H3N2) subclade K (J.2.4.1) has dominated the 2025/26 season start in England. Post-infection ferret antisera raised against northern hemisphere 2025/26 vaccine strains showed reduced reactivity to subclade K viruses in England, aligning with World Health Organization reports. Nevertheless, early post-vaccination, vaccine effectiveness against influenza-related emergency department attendances and hospital admissions remained within typical ranges, at 72–75% in children and adolescents (< 18 years) and 32–39% in adults. Hence, vaccination remains effective against clinical disease caused by influenza A(H3N2) viruses.

At the end of the southern hemisphere (SH) 2025 influenza season and the start of the northern hemisphere (NH) 2025/26 season, a rapid increase in influenza A(H3N2) subclade K (formerly J.2.4.1) incidence was observed, with subclade K projected to dominate among H3N2 viruses during the 2025/26 season [1-3]. This marks a notable A(H3N2) virus evolution since the NH 2025/26 vaccine strains were selected (based on the J.2 subclade). Subclade K viruses are characterised by J.2.4 subclade defining mutations T135K and K189R, as well as additional ones including K2N, S144N, N158D, I160K, Q173R, T328A and S378N [2-5]. Early analysis from the September 2025 World Health Organization (WHO) vaccine composition meeting suggested low reactivity of subclade K viruses with post-infection ferret antisera raised against the NH vaccine strains [6]. It is unclear how 2025/26 vaccine effectiveness (VE) against clinical disease may be affected. Here, we report the genetic and antigenic characterisation of H3N2 viruses in England and VE against influenza emergency department (ED) attendance and hospital admission.

## Epidemiological context

Influenza activity in England began unusually early in the 2025/26 season, with rises in influenza-like-illness ED attendances and test positivity in children and adolescents (aged < 18 years) and young adults (aged 18–24 years) [7]. Most all-age influenza indicators, including influenza hospitalisations and intensive care unit admissions, went above baseline levels from week 43 2025 (which started on 20 October), and this represented the earliest inter-pandemic start to the season in England since 2003/04 [7]. Most cases (98%) since week 40 have been influenza A and, where subtyping was available, 95% (children and adolescents), 84% (adults aged 18–64) and 65% (adults aged ≥ 65 years) were A(H3N2), as described in Supplementary Figure 1. Early starts to the 2025/26 season have also been observed elsewhere in the NH [8,9].

## England’s vaccination programme

England’s vaccination programme is based predominantly on enhanced vaccines (not standard-dose egg-based vaccines) (Table 1) [10]. Standard-dose egg-based vaccines are only recommended where other vaccines are unavailable. Vaccine uptake for this season’s campaigns was 34% (2–3-year-olds), 29% (6 months to < 65 years in a clinical risk group) and 62% (adults aged ≥ 65 years) up to 2 November (uptake data unavailable for school-aged children and adolescents not in a clinical risk group) [7].

**Table 1:** Overview of influenza vaccination programme, England, 2025/26 season

Age or risk group	First-line vaccine	Second-line vaccine
≥ 65 years	aIIV, IIV-HD, IIVr	IIVc
60–64 years (risk group)	IIVc, IIVr, aIIV, IIV-HD	IIVe
50–64 years (risk group)	IIVc, IIVr, aIIV	IIVe
18–64 years (risk group)	IIVc, IIVr	IIVe
2–17 years	LAIV	IIVc
2–17 years (unable to have LAIV)	IIVc	IIVe
6 months – < 2 years (risk group)	IIVc	IIVe

aIIV: adjuvanted vaccine; IIV-HD: high-dose vaccine; IIVc: cell-based vaccine, IIVe: egg-based vaccine; IIVr: recombinant vaccine; LAIV: live-attenuated influenza vaccine.

## Influenza genetic characterisation

As detailed in the Supplementary Appendix, Genetic and Antigenic Characterisation Methods subsection, genetic characterisation by whole genome sequencing, was performed on influenza viruses detected via primary care sentinel surveillance and those received in the national reference laboratory in Colindale, London, from secondary care referrals between week 10 and week 43. A predominance of subclade K among A(H3N2) viruses was observed since week 35 2025, with between this week and week 43, 156 viruses of this subclade (87%) found among 179 A(H3N2) viruses (Table 2). The distribution of all genetically characterised influenza A and B detections since week 10 2025 is illustrated in Supplementary Figure 2a and the phylogenetic analysis of A(H3N2) sequences in Supplementary Figure 2b.

**Table 2:** Influenza A(H3N2) viruses genetically characterised by whole genome sequencing, England, week 10^a^–week 43 2025 (n = 281 detections)

Type	Subtype	Clade	Subclade	2025 Range of weeks (dates)			Number of detections
				Week 10−20 (03 Mar−18 May)	Week 21−34 (19 May−24 Aug)	Week 35 − 43 (25 Aug−20 Oct)	
A	H3N2	2a.3a.1	J	1	0	0	1
A	H3N2	2a.3a.1	J.1.1	2	0	0	2
A	H3N2	2a.3a.1	J.2	63	6	11	80
A	H3N2	2a.3a.1	J.2.2	25	1	0	26
A	H3N2	2a.3a.1	J.2.3	0	1	0	1
A	H3N2	2a.3a.1	J.2.4	0	1	11	12
A	H3N2	2a.3a.1	K(J.2.4.1)	0	2	156	158
A	H3N2	2a.3a.1	J.2.5	0	0	1	1
**Total**				**91**	**11**	**179**	**281**

^a^ Week 10 2025 starts on 3 March, as all weeks in the table start on a Monday.

## Influenza A(H3N2) antigenic characterisation

**Figure 1:**
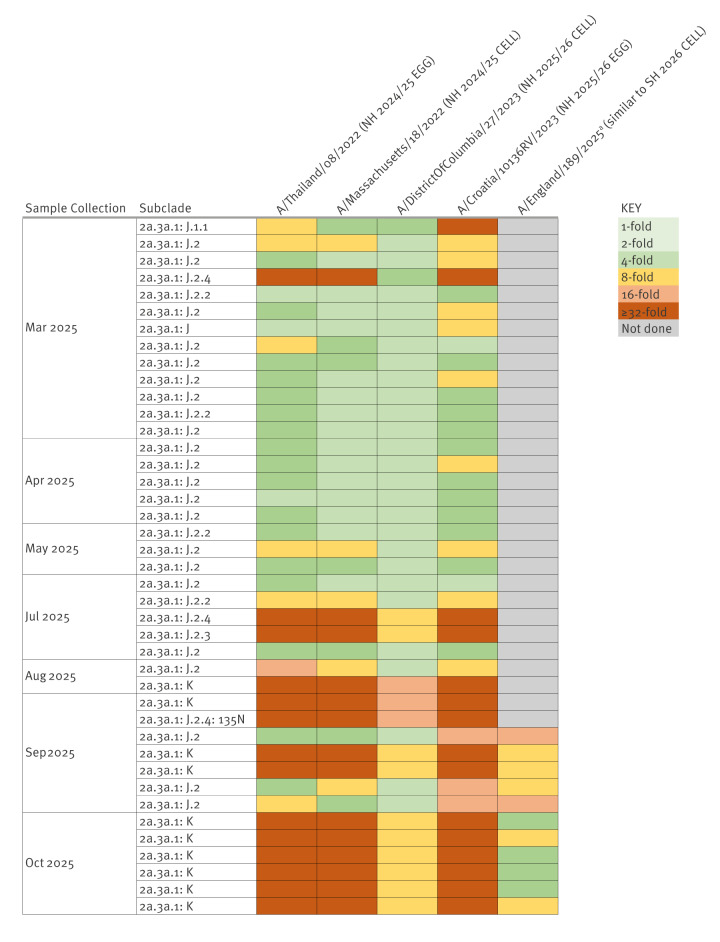
Influenza A(H3N2) viruses antigenically characterised by haemagglutination inhibition (HAI) assay, England, March–October 2025 (n = 41 viruses)

With approaches described in the Supplementary Appendix, Genetic and Antigenic Characterisation Methods subsection, antigenic characterisation by haemagglutination inhibition (HAI) assay of 41 A(H3N2) influenza viruses collected between March and October 2025 with antisera raised against current vaccine strains found a tendency towards reduced reactivity over time, consistent with the observed genetic diversification (Figure 1). Most viruses from the J.2 and J.2.2 subclades reacted well, with a majority reacting within 4-fold of the current vaccine strain homologous titres. Viruses in subclade J.2.3, J.2.4 and J.2.4 (+ 135N) were low reactors (i.e. reacting less than or equal to 8-fold) with ferret antisera raised against NH 2025/26 vaccine strains (Figure 1). All 10 subclade K viruses isolated between August and October 2025 showed a > 32-fold reduction in reactivity with ferret antisera raised against egg-propagated A/Croatia/10136RV/2023 and at least eightfold reduction with antisera raised against cell-propagated A/District of Columbia/27/2023. Ferret antisera raised against A/England/189/2025 (a virus similar to the SH 2026 vaccine strain: A/Sydney/1359/2024-like virus) recognised viruses from K subclade moderately well, with 4/8 viruses reacting within fourfold of the homologous titre.

HAI: haemagglutination inhibition.

^a^ An A/Sydney/1359/2024-like virus.

Individual clinical virus isolates from England are defined by month of sample collection, genetic subclade and reactivity with post-infection ferret antisera. Key indicates fold change in HAI titre achieved by clinical virus isolates compared with the homologous titre for the post-infection ferret antisera for the vaccine and reference virus strains.

## Vaccine effectiveness against influenza A and influenza A(H3N2)

A test-negative case–control (TNCC) study design was used to estimate VE against influenza [11-14]. Individuals for the TNCC were found in a database called the Emergency Care Data Set (ECDS), which is further described in the Vaccine Effectiveness Methods (data sources subsection) in the Supplementary Appendix. In the ECDS, candidates for the TNCC were identified as patients who attended the ED, or were admitted to hospital, and who additionally had undergone a PCR test for influenza at any time between 14 days before their ED/hospital visit to up to 2 days after. Cases were individuals attending ED or admitted to hospital with influenza positive PCR tests, and controls were comparable individuals with influenza negative PCR tests. Individuals were considered vaccinated if their PCR test was performed 14 days or more after receiving an influenza vaccine.

Multivariable logistic regression was used with the test result as the outcome, vaccination status as the exposure of interest and with confounder adjustment for test week, age, region and clinical risk status. As explained in the Supplementary Vaccine Effectiveness Methods, VE was estimated against ED attendance and hospital admission stratified by age group and by influenza type and subtype.

The main analysis included tests from 29 September to 2 November 2025. The temporal distribution of cases and controls is shown in Supplementary Figure 3, and descriptive characteristics are presented in Supplementary Tables 1–6.

**Figure 2:**
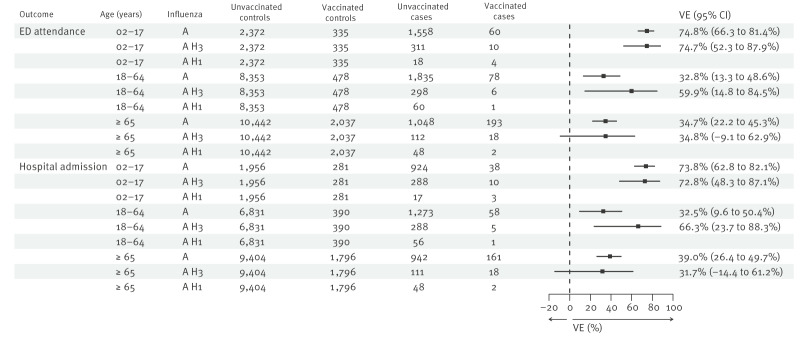
Vaccine effectiveness against emergency department attendance and hospital admission for children and adolescents aged 2 to 17 years, adults aged 18 to 64 years and adults aged ≥ 65 years, England, 29 September to 2 November 2025 (n = 28,789 cases and controls)

CI: confidence interval; ED: emergency department; VE: vaccine effectiveness.

Among children and adolescents aged 2 to 17 years, VE against ED attendance and admission with influenza A and influenza A(H3N2) was high with point estimates at around 72–75% (Figure 2). In all adults, moderate VE (32–39%) was observed against ED attendance and admission with influenza A. Point estimates against influenza A(H3N2) were higher for adults aged 18–64 years at around 60%, but with wide and overlapping confidence intervals, while A(H3N2) estimates were similar to influenza A estimates for those aged ≥ 65 years. Three sensitivity analyses found similar results, with the first restricting ED attendance and hospital admissions to respiratory-coded reasons, as presented in Supplementary Figure 4. The second sensitivity analysis used a different source of hospitalisation data (the Secondary Uses Service (SUS)), as shown in Supplementary Figure 5. The third restricted the study period to 13 October to 2 November 2025, as illustrated in Supplementary Figure 6. This was because most adults in England were eligible for vaccination from 1 October and individuals were required to be vaccinated for at least 14 days to be included in the TNCC as ‘vaccinated’; hence, the first 2 weeks of the main study period included very few fully vaccinated adults.

There were insufficient case numbers of influenza A(H1N1) to estimate VE.

## Discussion

In line with WHO reports [6], we also found reduced reactivity of subclade K viruses with post-infection ferret antisera raised against the NH 2025/26 vaccine strains. Nevertheless, we find reassuring early evidence that a programme using NH-strain enhanced vaccines offers protection against clinical influenza disease. Protection was highest among children and adolescents, where VE was 72–75%. In adults, VE was lower with most estimates around 32–39%. This is similar to end of season VE against influenza A(H3N2) in recent years in the United Kingdom, Europe and Canada [11,12,15-19].

Our high VE results in children and adolescents are consistent with some previous LAIV studies with antigenically drifted H3N2 viruses [20-22], but not all [23]. The apparent cross-protection against drifted viruses may be a result of the breadth of the immune response provided by LAIV. Considering the English vaccination programme, our own antigenic analysis indicates that the reduction in reactivity is greater with antisera raised against the NH 2025/26 egg strain compared with the cell strain. The widespread use of enhanced (including non-egg-based) vaccines in adults in England may have helped maintain VE.

Our results are encouraging, though the study reflects a period soon after vaccination, before any waning in effectiveness. Recent end-of-season analyses have highlighted within-season waning in adults [24] and it will be important to monitor duration of protection this year. We explored the possibility of healthy vaccinee bias by looking for a vaccine effect in the 0–6-day period post-vaccination (before an effect would be expected), however, there was no evidence of an effect during this period, as depicted in Supplementary Figure 7. In children and adolescents, we found negative VE which is likely due to detections of LAIV strains soon after vaccination (Supplementary Figure 7).

To date, disease burden has been greatest in children, adolescents and young adults in England [7]. Lower VE in adults is consistent with previous seasons but may also reflect higher pre-existing immunity against the circulating strains, reducing the additional benefit of vaccination. Seroprevalence analyses will be needed to confirm this.

Since influenza testing in most ED departments is targeted at those with acute respiratory infection symptoms, to maximise statistical power, all ED attendances and hospitalisations temporally associated with an influenza test were included [13]. Sensitivity analyses restricting to those with a respiratory code found similar estimates. We used ED and hospital data from ECDS in the primary analysis because it is less lagged than SUS data used in previous studies, but sensitivity analyses using SUS produced similar estimates.

Due to the small proportion of subtyped tests, VE estimates against A(H3N2) had wide confidence intervals. In children and adolescents, where 95% of subtyped viruses were A(H3N2), overall influenza A results very likely reflect VE against A(H3N2). In adults, 84% and 65% of subtyped viruses were A(H3N2) in ages 18–64 and ≥ 65 years, so we can be less confident in older adults especially that influenza A results reflect A(H3N2) results. Nonetheless, point-estimates against A(H3N2) were similar if not higher than overall influenza A results in these cohorts. Results in adults aged ≥ 65 years were similar to the overall influenza A results, whereas in adults aged 18–64 years point-estimates were higher for A(H3N2). This was not statistically significant and most likely due to small case numbers. As the season continues, we will continue to monitor this. Furthermore, case numbers are currently too low to estimate effectiveness by vaccine type, but this will also be important to assess.

Most characterised H3N2 viruses were subclade K, though it is also noted that secondary care samples referred to the national reference laboratory for viral characterisation are dependent on adherence to referral practices as described in [25]. Samples from some regions are received in batches therefore data in early season may not be geographically representative. However, primary care samples are collected from sentinel practices distributed across England and all samples tested in real-time (Supplementary Figure 2a).

## Conclusion

Despite the emergence of a drifted influenza A(H3N2) strain driving an unusually early 2025/26 NH influenza season, our early estimates provide reassurance that current NH enhanced vaccines provide protection in children, adolescents and adults in the early period post-vaccination. The high VE in children and adolescents strengthens the case for optimising vaccine uptake in this group, where we could also see indirect protection of other age cohorts [26].

## Data Availability

All sequence data is publicly available on GISAID. Since the vaccine effectiveness work is carried out under Regulation 3 of The Health Service (Control of Patient Information; Secretary of State for Health, 2002) using patient identification information without individual patient consent as part of the UKHSA legal requirement for public health surveillance and monitoring of vaccines, authors cannot make the underlying dataset publicly available for ethical and legal reasons. However, all the data used for this analysis are included as aggregated data in the manuscript tables and appendix. Applications for relevant anonymised data should be submitted to the UKHSA Office for Data Release: https://www.gov.uk/government/publications/accessing-ukhsa-protected-data.

## Supplementary Data

1849000 SupplementaryMaterial
