# Supplementary material for: Early influenza virus characterisation and vaccine effectiveness in England in autumn 2025, a period dominated by influenza A(H3N2) subclade K
Source: Euro Surveill. 2025 Nov 20;30(46):2500854. doi: 10.2807/1560-7917.ES.2025.30.46.2500854 (PMC12639273; doi:10.2807/1560-7917.ES.2025.30.46.2500854)

Supplementary Appendix

Early influenza virus characterisation and vaccine effectiveness in England in autumn 2025, a period dominated by influenza A(H3N2) subclade K

This supplementary material is hosted by *Eurosurveillance* as supporting information alongside the article 'Early influenza virus characterisation and vaccine effectiveness in England in autumn 2025, a period dominated by influenza A(H3N2) subclade K', on behalf of the authors, who remain responsible for the accuracy and appropriateness of the content. The same standards for ethics, copyright, attributions and permissions as for the article apply. Supplements are not edited by *Eurosurveillance* and the journal is not responsible for the maintenance of any links or email addresses provided therein.

Supplementary Figure 1.

Surveillance data from the Respiratory Datamart surveillance system in England[1] (covering the national reference laboratory, regional public health laboratories and sentinel hospital laboratories), showing the number and proportion of influenza tests by subtype amongst individuals aged 2 to 17, 18 to 64 and 65+.

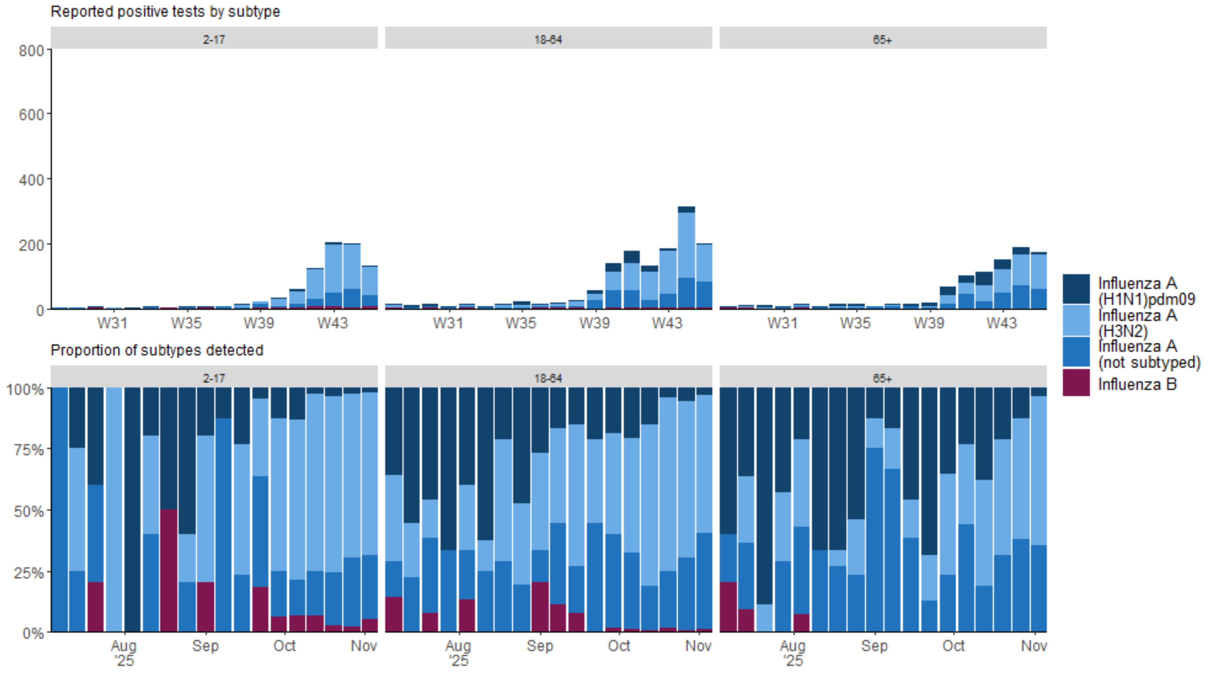

[1] https://www.gov.uk/government/publications/sources-of-surveillance-data-for-influenza-covid-19-and-other-respiratory-viruses/data-quality-report-national-flu-and-covid-19-surveillance-report#laboratory-surveillance

Supplementary Genetic and Antigenic Characterisation Methods

Influenza virus genetic and antigenic characterisation in England is undertaken at the Respiratory Virus Unit (RVU) national reference laboratory and is comprised of two main data sources; primary care and secondary care virological surveillance.

Primary care sentinel surveillance is undertaken via a subset of Oxford-Royal College of General Practitioners Research and Surveillance Centre (RCGP-RSC) network practices (around 300) that participate in weekly virology surveillance. Practices collect nasopharyngeal samples from patients presenting to their general practitioner (GP) with symptoms of any acute respiratory infection or influenza-like illness with an onset date within the last 10 days. Samples are transported to the RVU reference laboratory for primary testing.

Secondary care surveillance occurs via UKHSA regional Clinical Network Laboratories and some NHS laboratories that refer influenza positive samples to RVU for further characterization, following published national guidance[2].

Influenza detection by RT-PCR and whole genome sequencing

Between week 10 to week 43 2025, all primary care and a proportion of referred secondary care samples were tested using in-house developed multiplexed real-time RT-PCR assays to determine influenza type and subtype. The study period spanned the late influenza 2024/25 season, the 2025 summer period (outside the season) and the early 2025/26 influenza virus season. Samples received in the Reference laboratory (from primary care sentinel surveillance and secondary care referrals) that test positive for influenza and meet pre-defined quality control criteria (e.g. Ct value) undergo genetic characterization by whole genome sequencing (WGS) using an in-house workflow. Outside the influenza season, all primary and secondary care surveillance samples that tested positive for influenza and with sufficient viral load are selected for WGS. Early and late in the season, once influenza positivity rate is >10%, additional selection criteria are applied. Samples prioritised for sequencing include those with severe illness, antiviral treatment, vaccination history, underlying comorbidities or part of outbreak investigation, Geographic location, age, gender representativeness are also considered. During the study period, the additional selection criteria was applied between week 10 and week 18.

WGS was performed as previously described[3]. Briefly, following RNA extraction, one-step RT-PCR was performed using influenza-specific primers[4]. Sequencing libraries were prepared for sequencing using the DNA Prep library preparation kit (Illumina) from generated amplicons and sequenced using short-read, Illumina sequencing technology. Reads were mapped with BWA v0.7.5 and converted to BAM files using SAMTools (1.1.2). Variants were called using QuasiBAM, an in-house developed script was used to analyze the consensus sequence for signature amino acid changes and phylogenetic clustering to determine the genetic clade/subclade.

Virus culture and antigenic characterisation

A representative subset of influenza viruses from clinical samples as well as influenza viruses with novel or unusual changes identified by genomic surveillance are selected for virus culture and

[2] https://www.gov.uk/government/publications/referring-influenza-samples-to-ukhsas-respiratory-virus-unit/referral-of-influenza-samples-to-rvu-ukhsa-colindale-2024-to-2025

[3] Goldhill DH, Langat P, Xie H, et al. Determining the Mutation Bias of Favipiravir in Influenza Virus Using Next-Generation Sequencing. J Virol. 2019;93(2):e01217-18. Published 2019 Jan 4. doi:10.1128/JVI.01217-18

[4] Zhou B, Donnelly ME, Scholes DT, George KS, Hatta M, Kawaoka Y, Wentworth DE. 2009. Single-reaction genomic amplification accelerates sequencing and vaccine production for classical and Swine origin human influenza a viruses. J Virol 83:10309–10313. doi: 10.1128/JVI.01109-09.

antigenic characterisation by haemagglutination inhibition (HAI) assay. HAI was performed using standard methods[5].

Applying a Sequence First approach, viruses were selected for culture and antigenic characterization from clinical samples received from primary and secondary care that tested positive by RT-PCR for influenza A(H1N1)pdm09, A(H3N2) or influenza B and had a minimum of haemagglutinin (HA) sequence available from whole genome sequencing. Co-infections with SARS-CoV-2 or other respiratory viruses were excluded.

Virus culture was performed at containment level 2 using MDCK and MDCK-SIAT cells. Safety criteria applied for selection of respiratory samples including testing negative by RT-PCR for SARS-CoV-2 and exclusion of samples with risk factors for processing at higher containment including co-infection.

Quality metrics included restriction to RT-PCR Ct values less than 31, appropriate sample collection not into lysis buffer, and collection within10 days of onset.

Samples were selected for culture ensuring all subclades and emerging mutations identified by whole genome sequencing were represented. Samples from patients who had received influenza vaccine and those who were not vaccinated were selected.

[5] WHO 2011. WHO global influenza surveillance network: manual for the laboratory diagnosis and virological surveillance of influenza. https://www.who.int/publications/i/item/manual-for-the-laboratory-diagnosis-and-virological-surveillance-of-influenza

Supplementary Vaccine Effectiveness Methods

Data Sources

Influenza testing data

Laboratory testing data were extracted on 7th November 2025. Two sources of laboratory PCR testing data were used to identify influenza positive cases and influenza negative controls in England. First, the Respiratory Datamart, a sentinel laboratory surveillance system, with 9 participating laboratories in the 2025/26 influenza season, and secondly, the Second-Generation Surveillance System (SGSS), which records laboratory outcomes across England. Testing from the Respiratory Datamart are largely a subset of those in SGSS, with additional influenza A subtype information available for some laboratories. Datamart and SGSS were also used to identify the influenza samples which had also been tested for COVID-19.

Immunisation Information System (IIS)

The testing data were linked to the UKHSA IIS (a national vaccine register containing vaccine histories and demographic information on the whole population of England registered with a GP), using combinations of the unique individual NHS number, date of birth, surname, first name, and postcode using deterministic linkage. The IIS was accessed for dates of influenza vaccination and vaccine type, demographic data including sex, date of birth, ethnicity, index of multiple deprivation (IMD) quintile (small area measures of relative deprivation based on postcode) and NHS region. Clinical risk group status (those identified as being eligible for an influenza vaccine by NHS Cohorting as a Service (CaaS) was also extracted.

Emergency Care Data Set (ECDS)

Testing and vaccination data were linked to England's national Emergency Care Data Set (ECDS) to identify ED attendances and hospital admissions ECDS is the national dataset for urgent and emergency care in England. It includes hospital admissions through emergency department but not elective admissions. Testing data were linked to ECDS using NHS number and date of birth to identify ED attendances and hospital admissions within -2 to 14 days of the test. Overall, 95.6% of tests were within 0 to -2 days of the ECDS arrival date, reflecting the fact that the majority of influenza PCR testing occurs in hospital settings in England. Admissions due to an injury were excluded. Admissions with the reason for attending emergency care being a SNOMED CT (Systematized Nomenclature of Medicine–Clinical Terms) coded acute respiratory illness were flagged. The study period for the ECDS analysis was 29 September to 02 November 2025.

Secondary Uses Service

A sensitivity analysis was also run using the Secondary Uses Service (SUS), the national electronic database of hospital admissions that provides timely updates of ICD-10 codes for completed hospital stays for all NHS hospitals in England. Testing data were linked to SUS using NHS number and date of birth. SUS was used to identify hospitalisations within 14 days of a respiratory swab, or where a swab was taken up to 2 days after admission. The study period for the SUS analysis was 29 September to 26 October 2025, to allow for more data lags than the ECDS dataset.

Exclusions

Tests from individuals aged 2 years and older (on 31st August 2025) and resident in England were included. Tests without a sample date were excluded, as were tests where the influenza status was not fully known (i.e. controls required both influenza A and B negative results). Testing data were first de-duplicated such that no more than one test per person per 28-day period was retained, and a new positive test was kept over a negative test so that any positive test around the time of a respiratory hospital admission defined a case. We then further restricted to inclusion of the first of each influenza

A(H1N1)pdm09, A(H3N2) and B positive test, and where influenza A was not subtyped, we ensured positive tests were at least 6 weeks apart. We further excluded tests with no linkage to IIS, within 0 to 13 days of vaccination, plus adults with a record of receiving LAIV and children that received a recombinant or adjuvanted vaccine. Given the association between COVID-19 and influenza vaccination and its impact on the vaccination status of non-cases, SARS-CoV-2 positive controls were removed.

Covariates and adjustment

Vaccination status was the primary exposure variable of interest in all analyses. Additionally, all analyses included adjustment for week of test date, age group (2-3, 4-6, 7-10, 11-15, 16-17, 18-34, 35-49, 50-64, 65-74, 75-84, 85+), region and clinical risk group status (encoded as a categorial variable with a level for all conditions other than immunosuppression and a level for immunosuppression (as defined in the Green book). We assessed sex, ethnicity and IMD as potential confounders but did not include these in the final model as they did not change the vaccine effect by more than 1% so their inclusion was not deemed necessary.

Statistical methods

Multivariable logistic regression was used with the test result as the outcome, vaccination status as the primary exposure variable of interest and with confounder adjustment as described above. VE was calculated as 1-odds ratio and given as a percentage. To compare estimates, statistical significance was concluded where 95% confidence intervals (CIs) did not overlap.

Sensitivity analyses were conducted restricting the ED attendances and hospital admissions from ECDS to those coded as an acute respiratory attendance, and estimating VE against hospitalisation using the SUS dataset. Sensitivity analyses were also done to estimate VE by time since vaccination to investigate healthy vaccinee effects in the early period post-vaccination (0-6 days, 7-13 days, 2-4 weeks, 5-8 weeks). In this analysis those vaccinated in the 0-13 day period prior to the test date were included. Since most adults were eligible for vaccination from the 1st October and individuals were required to be vaccinated for at least 14 days to be included as vaccinated, the first two weeks of the main study period included very few fully vaccinated adults. We therefore also undertook a sensitivity analysis where we restricted the study period to week 42 to 44 (13 October to 2 November 2025).

Supplementary Figure 2.

Supplementary Figure 2a. Clade distribution of influenza A and B viruses in primary care sentinel surveillance in England, March to October 2025.

Samples were received from primary care sentinel surveillance as described in the Supplementary methods.

Late in the 2024/25 influenza season - the period from week 10 to week 20/2025 - Influenza B viruses dominated the detections with 63% of circulating viruses belonging to the B/Victoria lineage, followed by detection of influenza H3N2 (20%) and Influenza A(H1N1)pdm09 viruses, which accounted for approximately 17% of circulating influenza viruses characterised. During early summer (after week 20 (May)), Influenza A(H1N1)pdm09 dominated (64% of all influenza detections) - these detections belonged to genetic clade 5a.2a.1, subclade D.3.1. Antigenic analysis of viruses showed that the majority of A(H1N1)pdm09 viruses (97%) were well inhibited by post-infection ferret antisera raised against egg-propagated A/Victoria/4897/2022-like and cell-propagated A/Wisconsin/67/2022-like viruses, representing the 2025/26 Northern Hemisphere (NH) vaccine strain components. Influenza B and A(H3N2) viruses were detected at equal levels (18% each). All genetically analysed influenza B viruses from this period were in the V1A.3a.2 clade of the B/Victoria lineage and reacted well with post-infection ferret antisera raised to cell-propagated B/Austria/1359417/2021 representative of the 2025/26 NH vaccine strain. Most A(H3N2) viruses from this period genetically belonged to the clade 2a.3a.1. (mostly in the J.2 subclade) and were well inhibited by post-infection ferret antisera raised against cell-propagated A/DistrictofColumbia/27/2023 and egg-propagated A/Croatia/10136RV/2023, the 2025/26 NH vaccine strain components. However, sporadic detections of samples from subclade J.2.3, J.2.4 and J.2.5. were also observed.

From week 32 (week beginning 4th of August)[6], an increasing proportion of influenza detections were A(H3N2) – amongst which the proportion of subclade K viruses increased noticeably from week 36. This change in composition of H3N2 viruses was accompanied with a trend towards reduced antigenic reactivity over time as shown in Figure 1 (main text).

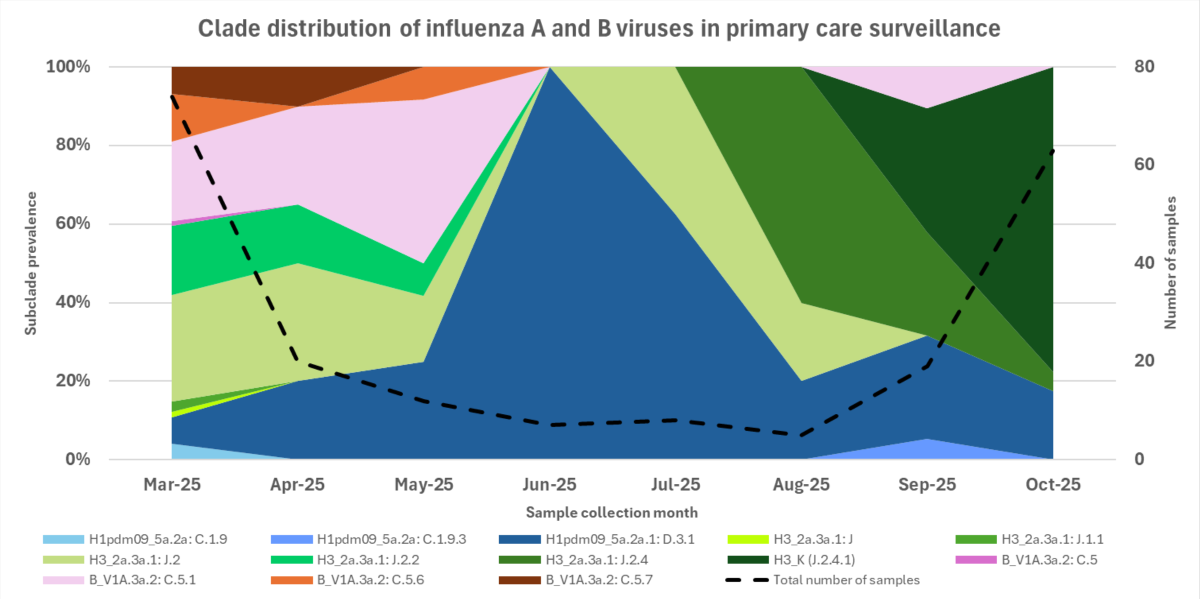

[6] https://www.gov.uk/government/statistics/national-flu-and-covid-19-surveillance-reports-2025-to-2026-season/national-flu-and-covid-19-surveillance-report-6-november-2025-week-45

Supplementary Figure 2b. Phylogenetic analysis of 278 influenza A(H3N2) full-length segment 4 (haemagglutinin) sequences collected between week 10 2025 and week 43 2025 with root EPI1857216 generated using NextClade [7,8]. UKHSA isolates are shown as balls at the tip among a subset of international strains as thin branches. Different subclades are shown in different colours. The X-axis represents genetic divergence.

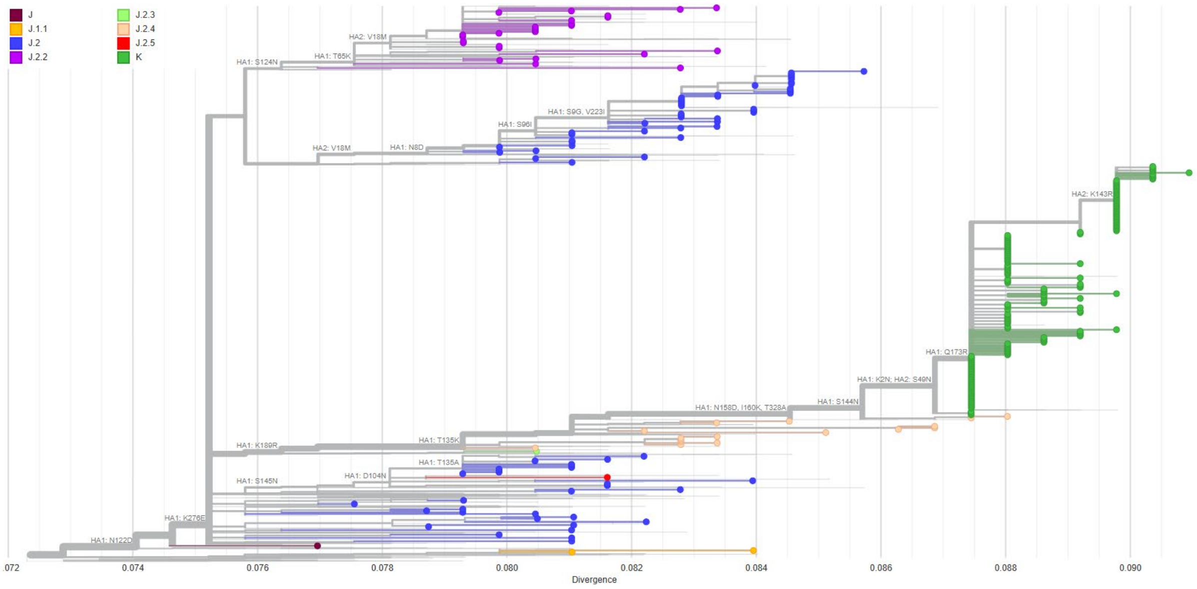

---

[7] https://clades.nextstrain.org

[8] Aksamentov, I., Roemer, C., Hodcroft, E. B., & Neher, R. A., (2021). Nextclade: clade assignment, mutation calling and quality control for viral genomes. Journal of Open Source Software, 6(67), 3773, https://doi.org/10.21105/joss.03773

Supplementary Figure 3.

Supplementary Figure 3a. The distribution of cases and controls over time during the study, amongst children aged 2 to 17 years, adults aged 18 to 64 years and adults aged 65 years and older.

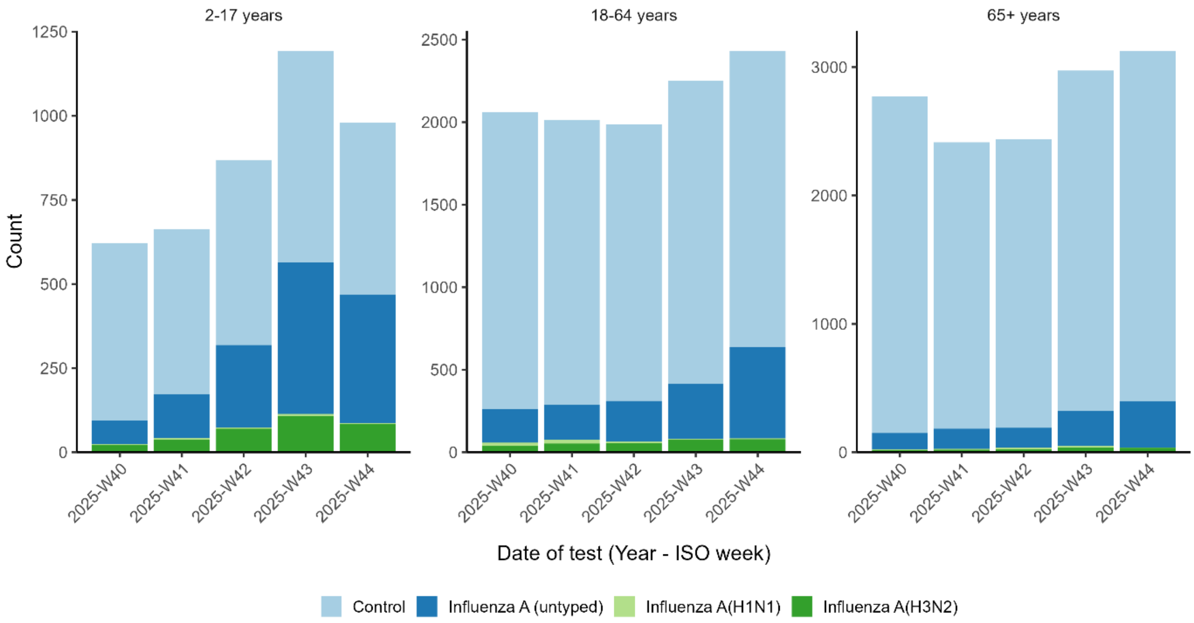

Supplementary Figure 3b. The distribution of influenza A cases and controls by vaccination status over time during the study, amongst children aged 2 to 17 years, adults aged 18 to 64 years and adults aged 65 years and older.

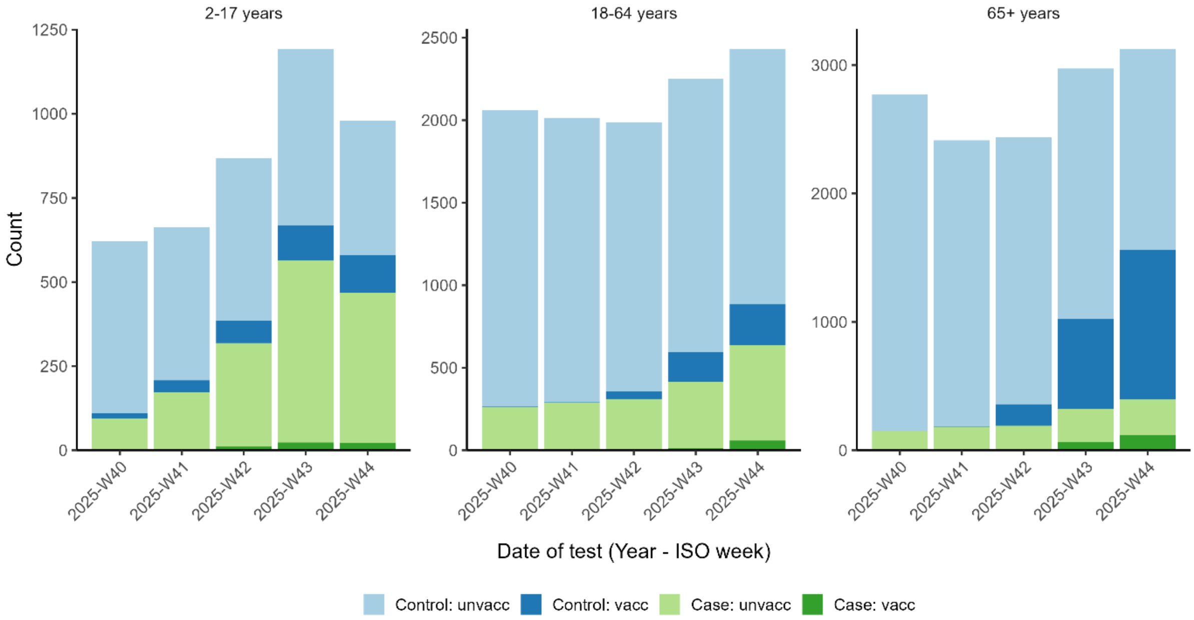

Supplementary Table 1

Descriptive characteristics of children aged 2 to 17 years included in the analysis estimating vaccine effectiveness against ED attendance.

Characteristic	Controls N = 2,707[1]	Influenza A N = 1,618[1]	Influenza A(H1N1) N = 22[1]	Influenza A(H3N2) N = 321[1]
Vaccination Status				
Unvaccinated	2,372 (88%)	1,558 (96%)	18 (82%)	311 (97%)
Vaccinated	335 (12%)	60 (3.7%)	4 (18%)	10 (3.1%)
Age (years)				
2-3	955 (35%)	393 (24%)	10 (45%)	78 (24%)
4-6	643 (24%)	447 (28%)	6 (27%)	78 (24%)
7-10	459 (17%)	264 (16%)	2 (9.1%)	47 (15%)
11-15	440 (16%)	404 (25%)	4 (18%)	98 (31%)
16-17	210 (7.8%)	110 (6.8%)	0 (0%)	20 (6.2%)
Region				
East Midlands	223 (8.2%)	183 (11%)		4 (1.2%)
East of England	182 (6.7%)	47 (2.9%)	3 (14%)	7 (2.2%)
London	304 (11%)	190 (12%)		14 (4.4%)
North East	353 (13%)	128 (7.9%)		1 (0.3%)
North West	641 (24%)	604 (37%)	6 (27%)	252 (79%)
South East	281 (10%)	111 (6.9%)		2 (0.6%)
South West	348 (13%)	62 (3.8%)	8 (36%)	13 (4.0%)
West Midlands	215 (7.9%)	179 (11%)	1 (4.5%)	22 (6.9%)
Yorkshire and Humber	160 (5.9%)	114 (7.0%)	4 (18%)	6 (1.9%)
Clinical Risk Status				
No risk	2,101 (78%)	1,335 (83%)	17 (77%)	256 (80%)
At risk; not immunosuppressed	468 (17%)	226 (14%)	2 (9.1%)	47 (15%)
At risk; immunosuppressed	138 (5.1%)	57 (3.5%)	3 (14%)	18 (5.6%)

[1] n (%)

Supplementary Table 2

Descriptive characteristics of adults aged 18 to 64 years included in the analysis estimating vaccine effectiveness against ED attendance.

Characteristic	Controls N = 8,831[1]	Influenza A N = 1,913[1]	Influenza A(H1N1) N = 61[1]	Influenza A(H3N2) N = 304[1]
Vaccination Status				
Unvaccinated	8,353 (95%)	1,835 (96%)	60 (98%)	298 (98%)
Vaccinated	478 (5.4%)	78 (4.1%)	1 (1.6%)	6 (2.0%)
Age (years)				
18-34	2,416 (27%)	976 (51%)	15 (25%)	175 (58%)
35-49	2,481 (28%)	436 (23%)	11 (18%)	75 (25%)
50-64	3,934 (45%)	501 (26%)	35 (57%)	54 (18%)
Region				
East Midlands	432 (4.9%)	203 (11%)	1 (1.6%)	7 (2.3%)
East of England	668 (7.6%)	89 (4.7%)	5 (8.2%)	20 (6.6%)
London	1,071 (12%)	258 (13%)	2 (3.3%)	25 (8.2%)
North East	1,147 (13%)	119 (6.2%)		2 (0.7%)
North West	1,677 (19%)	542 (28%)	14 (23%)	178 (59%)
South East	829 (9.4%)	111 (5.8%)	2 (3.3%)	2 (0.7%)
South West	1,212 (14%)	129 (6.7%)	21 (34%)	10 (3.3%)
West Midlands	1,064 (12%)	208 (11%)	10 (16%)	51 (17%)
Yorkshire and Humber	731 (8.3%)	254 (13%)	6 (9.8%)	9 (3.0%)
Clinical Risk Status				
No risk	4,082 (46%)	1,118 (58%)	24 (39%)	199 (65%)
At risk; not immunosuppressed	3,860 (44%)	665 (35%)	33 (54%)	85 (28%)
At risk; immunosuppressed	889 (10%)	130 (6.8%)	4 (6.6%)	20 (6.6%)

[1] n (%)

Supplementary Table 3

Descriptive characteristics of adults aged 65 years and older included in the analysis estimating vaccine effectiveness against ED attendance.

Characteristic	Controls N = 12,479[1]	Influenza A N = 1,241[1]	Influenza A(H1N1) N = 50[1]	Influenza A(H3N2) N = 130[1]
Vaccination Status				
Unvaccinated	10,442 (84%)	1,048 (84%)	48 (96%)	112 (86%)
Vaccinated	2,037 (16%)	193 (16%)	2 (4.0%)	18 (14%)
Age (years)				
65-74	3,882 (31%)	448 (36%)	12 (24%)	51 (39%)
75-84	4,964 (40%)	483 (39%)	24 (48%)	41 (32%)
85+	3,633 (29%)	310 (25%)	14 (28%)	38 (29%)
Region				
East Midlands	546 (4.4%)	128 (10%)	1 (2.0%)	3 (2.3%)
East of England	999 (8.0%)	52 (4.2%)	2 (4.0%)	5 (3.8%)
London	1,040 (8.3%)	173 (14%)		17 (13%)
North East	1,654 (13%)	57 (4.6%)		
North West	2,073 (17%)	332 (27%)	12 (24%)	53 (41%)
South East	1,267 (10%)	75 (6.0%)	1 (2.0%)	2 (1.5%)
South West	2,029 (16%)	114 (9.2%)	22 (44%)	11 (8.5%)
West Midlands	1,773 (14%)	160 (13%)	12 (24%)	39 (30%)
Yorkshire and Humber	1,098 (8.8%)	150 (12%)		
Clinical Risk Status				
No risk	2,004 (16%)	212 (17%)	10 (20%)	18 (14%)
At risk; not immunosuppressed	8,855 (71%)	886 (71%)	32 (64%)	91 (70%)
At risk; immunosuppressed	1,620 (13%)	143 (12%)	8 (16%)	21 (16%)

[1] n (%)

Supplementary Table 4

Descriptive characteristics of children aged 2 to 17 years included in the analysis estimating vaccine effectiveness against hospital admission.

	Controls	Influenza A	Influenza A(H1N1)	Influenza A(H3N2)
Characteristic	N = 2,237[1]	N = 962[1]	N = 20[1]	N = 298[1]
Vaccination Status				
Unvaccinated	1,956 (87%)	924 (96%)	17 (85%)	288 (97%)
Vaccinated	281 (13%)	38 (4.0%)	3 (15%)	10 (3.4%)
Age (years)				
2-3	789 (35%)	240 (25%)	9 (45%)	70 (23%)
4-6	525 (23%)	259 (27%)	5 (25%)	70 (23%)
7-10	398 (18%)	151 (16%)	2 (10%)	44 (15%)
11-15	364 (16%)	248 (26%)	4 (20%)	94 (32%)
16-17	161 (7.2%)	64 (6.7%)	0 (0%)	20 (6.7%)
Region				
East Midlands	150 (6.7%)	83 (8.6%)		4 (1.3%)
East of England	176 (7.9%)	41 (4.3%)	3 (15%)	7 (2.3%)
London	242 (11%)	90 (9.4%)		6 (2.0%)
North East	271 (12%)	78 (8.1%)		1 (0.3%)
North West	590 (26%)	397 (41%)	6 (30%)	240 (81%)
South East	219 (9.8%)	70 (7.3%)		1 (0.3%)
South West	306 (14%)	41 (4.3%)	7 (35%)	12 (4.0%)
West Midlands	169 (7.6%)	108 (11%)	1 (5.0%)	22 (7.4%)
Yorkshire and Humber	114 (5.1%)	54 (5.6%)	3 (15%)	5 (1.7%)
Clinical Risk Status				
No risk	1,704 (76%)	752 (78%)	15 (75%)	233 (78%)
At risk; not immunosuppressed	407 (18%)	162 (17%)	2 (10%)	47 (16%)
At risk; immunosuppressed	126 (5.6%)	48 (5.0%)	3 (15%)	18 (6.0%)

[1] n (%)

Supplementary Table 5

Descriptive characteristics of adults aged 18 to 64 years included in the analysis estimating vaccine effectiveness against hospital admission.

Characteristic	Controls N = 7,221[1]	Influenza A N = 1,331[1]	Influenza A(H1N1) N = 57[1]	Influenza A(H3N2) N = 293[1]
Vaccination Status				
Unvaccinated	6,831 (95%)	1,273 (96%)	56 (98%)	288 (98%)
Vaccinated	390 (5.4%)	58 (4.4%)	1 (1.8%)	5 (1.7%)
Age (years)				
18-34	1,878 (26%)	630 (47%)	13 (23%)	172 (59%)
35-49	1,982 (27%)	302 (23%)	9 (16%)	70 (24%)
50-64	3,361 (47%)	399 (30%)	35 (61%)	51 (17%)
Region				
East Midlands	344 (4.8%)	165 (12%)	1 (1.8%)	7 (2.4%)
East of England	625 (8.7%)	73 (5.5%)	5 (8.8%)	20 (6.8%)
London	796 (11%)	134 (10%)		14 (4.8%)
North East	893 (12%)	81 (6.1%)		2 (0.7%)
North West	1,349 (19%)	377 (28%)	14 (25%)	178 (61%)
South East	718 (9.9%)	84 (6.3%)	2 (3.5%)	2 (0.7%)
South West	965 (13%)	96 (7.2%)	21 (37%)	10 (3.4%)
West Midlands	971 (13%)	162 (12%)	10 (18%)	51 (17%)
Yorkshire and Humber	560 (7.8%)	159 (12%)	4 (7.0%)	9 (3.1%)
Clinical Risk Status				
No risk	3,147 (44%)	698 (52%)	21 (37%)	191 (65%)
At risk; not immunosuppressed	3,312 (46%)	530 (40%)	32 (56%)	82 (28%)
At risk; immunosuppressed	762 (11%)	103 (7.7%)	4 (7.0%)	20 (6.8%)

[1] n (%)

Supplementary Table 6

Descriptive characteristics of adults aged 65 years and older included in the analysis estimating vaccine effectiveness against hospital admission.

Characteristic	Controls N = 11,200[1]	Influenza A N = 1,103[1]	Influenza A(H1N1) N = 50[1]	Influenza A(H3N2) N = 129[1]
Vaccination Status				
Unvaccinated	9,404 (84%)	942 (85%)	48 (96%)	111 (86%)
Vaccinated	1,796 (16%)	161 (15%)	2 (4.0%)	18 (14%)
Age (years)				
65-74	3,481 (31%)	386 (35%)	12 (24%)	51 (40%)
75-84	4,442 (40%)	434 (39%)	24 (48%)	41 (32%)
85+	3,277 (29%)	283 (26%)	14 (28%)	37 (29%)
Region				
East Midlands	495 (4.4%)	123 (11%)	1 (2.0%)	3 (2.3%)
East of England	980 (8.8%)	48 (4.4%)	2 (4.0%)	5 (3.9%)
London	961 (8.6%)	151 (14%)		16 (12%)
North East	1,144 (10%)	33 (3.0%)		
North West	1,843 (16%)	279 (25%)	12 (24%)	53 (41%)
South East	1,176 (11%)	72 (6.5%)	1 (2.0%)	2 (1.6%)
South West	1,896 (17%)	106 (9.6%)	22 (44%)	11 (8.5%)
West Midlands	1,700 (15%)	156 (14%)	12 (24%)	39 (30%)
Yorkshire and Humber	1,005 (9.0%)	135 (12%)		
Clinical Risk Status				
No risk	1,781 (16%)	182 (17%)	10 (20%)	18 (14%)
At risk; not immunosuppressed	7,966 (71%)	786 (71%)	32 (64%)	90 (70%)
At risk; immunosuppressed	1,453 (13%)	135 (12%)	8 (16%)	21 (16%)

[1] n (%)

Supplementary Figure 4.

Sensitivity analysis estimating vaccine effectiveness by influenza type and subtype, restricting to respiratory coded ED attendances and hospital admissions.

Outcome	Age (years)	Influenza	Unvacc controls	Vacc controls	Unvacc cases	Vacc cases	VE (95% CI)
ED attendance: respiratory	02-17	Flu A	1094	180	937	34	77.7% (67 to 85.3%)
	02-17	Flu A H3	1094	180	173	7	71.8% (37.7 to 88.9%)
	02-17	Flu A H1	1094	180	11	3	
	18-64	Flu A	3460	212	1171	47	39.4% (14.8 to 57.6%)
	18-64	Flu A H3	3460	212	171	3	60.9% (-9.5 to 90.6%)
	18-64	Flu A H1	3460	212	38	1	
	65+	Flu A	5315	1041	723	131	41.1% (27 to 52.8%)
	65+	Flu A H3	5315	1041	64	12	
	65+	Flu A H1	5315	1041	28	2	
Hospital admission: respiratory	02-17	Flu A	858	148	511	21	76.2% (61.5 to 85.9%)
	02-17	Flu A H3	858	148	165	7	70.8% (34.7 to 88.7%)
	02-17	Flu A H1	858	148	10	3	
	18-64	Flu A	2870	175	803	35	39.9% (11.4 to 60.2%)
	18-64	Flu A H3	2870	175	166	3	59.7% (-15.1 to 90.5%)
	18-64	Flu A H1	2870	175	38	1	
	65+	Flu A	4894	952	655	114	44.5% (30.3 to 56.1%)
	65+	Flu A H3	4894	952	64	12	
	65+	Flu A H1	4894	952	28	2	

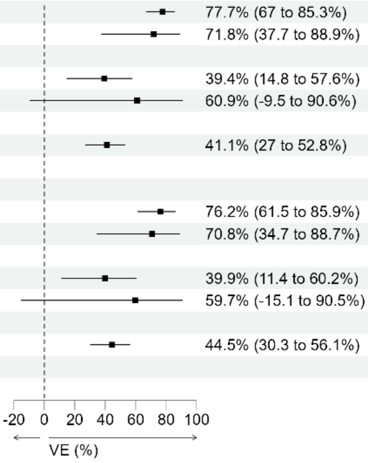

Supplementary Figure 5.

Sensitivity analysis estimating vaccine effectiveness against hospital admissions using the Secondary Use Services (SUS) dataset.

Outcome	Age (years)	Influenza	Unvacc controls	Vacc controls	Unvacc cases	Vacc cases	VE (95% CI)
SUS hosp	02-17	Flu A	2021	193	615	16	76.6% (61.2 to 86.8%)
	02-17	Flu A H3	2021	193	108	1	89.9% (52.8 to 99.4%)
	02-17	Flu A H1	2021	193	12	2	
	18-64	Flu A	7926	345	896	27	47.8% (22.7 to 66.1%)
	18-64	Flu A H3	7926	345	140	5	44.9% (-25.5 to 80.9%)
	18-64	Flu A H1	7926	345	52	2	
	65+	Flu A	10250	914	746	60	36.8% (16.1 to 53.1%)
	65+	Flu A H3	10250	914	82	9	
	65+	Flu A H1	10250	914	43	1	

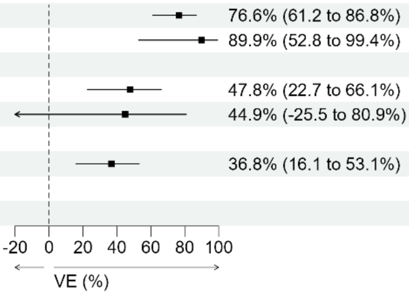

Supplementary Figure 6.

Sensitivity analysis estimating vaccine effectiveness by time since vaccination against ED attendance and admission.

Outcome	Age (years)	Influenza	Time since vacc	Unvacc controls	Vacc controls	Unvacc cases	Vacc cases	VE (95% CI)
ED attendance	02-17	Flu A	0-6d	2372	43	1558	56	-179.9% (-336.3 to -81%)
	02-17	Flu A	7-13d	2372	88	1558	19	62.2% (37.6 to 78.1%)
	02-17	Flu A	2-4w	2372	311	1558	55	74.4% (65.5 to 81.3%)
	02-17	Flu A	5-8w	2372	24	1558	5	78.9% (46.6 to 93.1%)
	18-64	Flu A	0-6d	8353	247	1835	45	-8.3% (-50.8 to 23.9%)
	18-64	Flu A	7-13d	8353	320	1835	39	31.6% (4.1 to 52.5%)
	18-64	Flu A	2-4w	8353	466	1835	76	32.3% (12.4 to 48.3%)
	18-64	Flu A	5-8w	8353	12	1835	2	56.1% (-70.7 to 93.4%)
	65+	Flu A	0-6d	10442	1086	1048	110	-3.4% (-27.3 to 16.7%)
	65+	Flu A	7-13d	10442	1289	1048	122	16.4% (-2 to 32%)
	65+	Flu A	2-4w	10442	2037	1048	193	35.6% (23.5 to 46%)
Hospital admission	02-17	Flu A	0-6d	1956	34	924	45	-270.8% (-510.1 to -128%)
	02-17	Flu A	7-13d	1956	70	924	14	53.3% (17.2 to 75.4%)
	02-17	Flu A	2-4w	1956	261	924	34	74.2% (62.9 to 82.6%)
	02-17	Flu A	5-8w	1956	20	924	4	70.4% (17.6 to 91.7%)
	18-64	Flu A	0-6d	6831	200	1273	33	-4.8% (-53.1 to 30.3%)
	18-64	Flu A	7-13d	6831	270	1273	33	23.1% (-11.1 to 48.4%)
	18-64	Flu A	2-4w	6831	380	1273	57	31.1% (7.5 to 49.6%)
	18-64	Flu A	5-8w	6831	10	1273	1	69% (-73.5 to 98.3%)
	65+	Flu A	0-6d	9404	968	942	95	2% (-22.4 to 22.3%)
	65+	Flu A	7-13d	9404	1134	942	106	17.7% (-1.8 to 34.1%)
	65+	Flu A	2-4w	9404	1796	942	161	39.9% (27.6 to 50.4%)

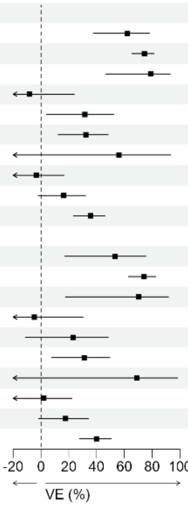

Supplementary Figure 7.

Sensitivity analysis estimating vaccine effectiveness, restricting the study period from week 42 to week 44 (13 October to 2 November 2025).

Outcome	Age (years)	Influenza	Unvacc controls	Vacc controls	Unvacc cases	Vacc cases	VE (95% CI)
ED attendance	02-17	Flu A	1406	284	1295	56	75.1% (66.4 to 81.9%)
	02-17	Flu A H3	1406	284	252	10	73.9% (50.6 to 87.6%)
	02-17	Flu A H1	1406	284	10	4	
	18-64	Flu A	4833	469	1288	78	33% (13.3 to 48.8%)
	18-64	Flu A H3	4833	469	204	6	61.2% (17.1 to 85.1%)
	18-64	Flu A H1	4833	469	19	1	
	65+	Flu A	5591	2035	716	192	35% (22.5 to 45.6%)
	65+	Flu A H3	5591	2035	77	18	35.6% (-7.8 to 63.4%)
	65+	Flu A H1	5591	2035	29	2	
Hospital admission	02-17	Flu A	1146	239	744	36	73.9% (62.4 to 82.3%)
	02-17	Flu A H3	1146	239	235	10	72.5% (47.6 to 87%)
	02-17	Flu A H1	1146	239	9	3	
	18-64	Flu A	3966	383	888	58	32.9% (9.9 to 50.9%)
	18-64	Flu A H3	3966	383	198	5	67.7% (26.3 to 88.8%)
	18-64	Flu A H1	3966	383	17	1	
	65+	Flu A	5098	1794	649	160	39.3% (26.7 to 50%)
	65+	Flu A H3	5098	1794	76	18	32.5% (-13.2 to 61.7%)
	65+	Flu A H1	5098	1794	29	2	

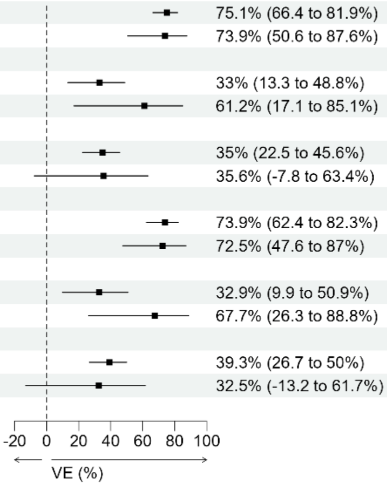

Supplement: SupplementaryMaterial [file 25-00854_SupplementaryAppendix.pdf]
